# The Influence of the Energy Intake Variability During the Week on the Body Composition in an Adult Population

**DOI:** 10.7150/ijms.92107

**Published:** 2024-06-11

**Authors:** Erika Čermáková, Martin Forejt, Martin Čermák

**Affiliations:** 1 Department of Public Health, Faculty of Medicine, Masaryk University, Kamenice 753/5, 625 00 Brno, Czech Republic; 2 Independent Researcher

**Keywords:** energy intake, variability in energy intake, percentage of body fat, BMI, 7-day food records, bioelectrical impedance

## Abstract

**Background:** The regularity of eating, together with other nutritional factors, is one of the important determinants of health. According to previous studies, it is not clear if a greater fluctuation in energy intake is associated with higher body fat and weight gain, or if the weight of people is stable despite these fluctuations in the energy intake. The aim of the study was to verify if a higher variability in the energy intake each day of the week is related to the amount of body fat and other anthropometric parameters.

**Methods:** A total of 220 (151 women, 69 men) individuals of Czech Caucasian origin with a BMI of 18.3-58 kg/m^2^, aged 21.7-79.7 were included in the study. Selected anthropometric characteristics were measured using a bioelectrical impedance analysis. 7-day food records were completed and analyzed using nutritional software. The measured values were statistically evaluated by multiple linear regression analysis.

**Results:** The results of the multiple linear regression showed the statistically significant dependence of the percentage of body fat (p<0.01), BMI (p<0.01), and waist circumference (p<0.05) on the relative variability of the daily energy intake.

**Conclusions:** The results of our study suggest that people with more regular energy intake also have better anthropometric parameters related to their cardiometabolic health.

## Introduction

Human nutrition is one of the most important lifestyle factors that influence one's health. The most important nutritional parameters that can reduce or increase the risk of a number of diseases include not only the quality and quantity of the diet, but also the regularity of the diet and the associated even and balanced energy intake on consecutive days.

In recent years, the regularity of eating and the associated variability of food energy intake has been studied most frequently in the context of intermittent fasting. These are various forms of restriction of calorie intake for a period of time within a few hours or a whole day (usually 12-24 hours). There are two main types: The first type of intermittent fasting is the complete restriction of intake of food every day for a defined period of the day (*time-restricted feeding*) 1. The second type restricts the energy intake on selected days of the week, most commonly every other day (*alternate-day fasting*) 2, or alternatively two days per week, the so-called 5:2 3, where fasting or a significant reduction in energy intake occurs at these defined times.

Intermittent fasting may be an effective tool for weight loss in people who are overweight or have obesity 1,4,5, but may also be associated with greater muscle loss compared with dietary interventions based on continuous caloric restriction 6.

Some types of studies that have looked at the variability in the energy intake have focused on the frequency of eating during the day in relation to the number of meals per day 7, skipping or including breakfast 8 or fasting for religious reasons 9. According to a recent meta-analysis and systematic review of studies, skipping breakfast may have a negative effect on the body composition or increase the risk of cardiovascular disease 10,11. However, according to some studies, the lower meal frequency may have a positive effect on weight loss 12.

Other types of studies related to dietary regularity have focused on the variability of food intake during the week. Most often, these types of studies focus on the variability of energy intake from day to day during the week 13 or related to the weekend 14 and the changes brought about by eating on holidays 15. Although weight fluctuations occur as a result of fluctuations in energy intake 16, studies to date have shown that it is unclear whether this irregularity in the diet is associated with a higher body fat percentage or weight gain 17,18 or whether people's weight is stable despite these fluctuations due to the corrective response of the body 19,20.

The aim of this study was to determine whether a greater variability in the energy intake across the days of the week in the case of eating *ad libitum* is associated with a higher body fat percentage and other parameters most commonly associated with cardiometabolic health.

## Methods

### Subjects

A total of 220 individuals were included in the study, 151 (68.6 %) women and 69 (31.4 %) men, BMI (31.7 ± 6.7), aged between 21.7 and 79.7 years (53 ± 14). Participants were approached through a mass media campaign aimed at residents of the South Moravian Region in the Czech Republic. Individuals with a pacemaker, insulin pump or cancer were excluded from the study. Before the measurement, all the participants were provided with background information about the study and subsequently signed an informed consent to participate in the research. The participants were anonymously coded and processed without any identifying data.

### Anthropometric characteristic

To determine the anthropometric characteristics of the subjects, basic anthropometric methods were used, such as the measurement of the body height and weight, and body composition. The body height was measured using a SECA 764 stadiometer (SECA GMBH & CO., Hamburg, Germany).

To measure the body composition, an InBody 230 device (Biospace, Seoul, Korea) that measures based on the principle of DSM-BIA (direct segmental multifrequency bioelectrical impedance analysis) and the software Lookin´Body basic (Biospace) and Lookin´Body 3.0 (Biospace) was used. The subjects should not have eaten or drunk anything before the measurement and should not have performed high-intensity physical activity (e.g., endurance running, swimming, strength training) for 2.5 hours. Before the measurements were taken, the subjects had an empty bladder. Adherence to the above guidelines was confirmed before each measurement. Among the anthropometric parameters studied, we included % body fat (BF), body mass index (BMI), waist circumference and waist-hip ratio (WHR).

The basal metabolic rate was assessed using the InBody 230 device, whose software uses predictive equations to estimate the final value based on the observed body composition results.

### Energy intake

Seven-day food records were used to determine the energy intake, in which the participants recorded data on all the foods and beverages consumed along with their amount. All the participants were first thoroughly instructed on how to record their food intake on pre-prepared forms in order to achieve the most accurate calculation of the energy intake. The food records were then analyzed with the NUTRIDAN II software to calculate the total amount of energy intake for each day of the week.

### Statistical analysis

Data were analyzed in Statistica version 12 (StatSoft) using multiple linear regression.

The significance level α was set at 5% (α = 0.05). Results with a p value <0.05 were considered statistically significant.

Linear regression was performed repeatedly for four anthropometric parameters: BMI, % body fat, waist circumference, and WHR. These anthropometric parameters were considered linearly dependent on the following independent parameters: height, age, basal metabolic rate (BMR), gender, mean energy intake and relative daily energy intake variability. The linear dependence of the anthropometric parameters on the independent parameters can be expressed as

y=b_0_+b_height_x_height_+b_age_x_age_+b_BMR_x_BMR_+b_gender_x_gender_+b_en.int._x_en.int_+b_var._x_var._

where y denotes the anthropometric parameters, x denotes the independent parameters, b_0_ is the constant coefficient, and b (with other subscripts) denotes the regression coefficients corresponding to each independent parameter. Except for gender, which is considered as a binary variable in the measurement, all the other variables are considered as non-binary variables. For the purpose of multiple linear regression, it was necessary to assign numerical values to both genders (male 0, female 1).

The absolute variability in the energy intake was determined from the standard deviation of the daily energy intakes. The relative variability in the energy intake was obtained by dividing the absolute variability of the energy intake by the average daily energy intake.

Linear regressions of all the anthropometric parameters (i.e., BMI, % BF, waist circumference and WHR) were also performed separately for men and women to compare the results. In this case, the sub-statistics worked with smaller data sets and with (one) fewer independent parameters (height, age, BMR, mean energy intake and relative daily energy intake variability). All the variables, in this case, are considered as non-binary variables. The equation describing the linear regression is almost identical to the previous one with the difference of omitting the "gender" index term.

The data corresponding to a given participant that did not contain a complete set of the aforementioned study parameters or a complete record of seven days of energy intake were excluded from the study data set. There were 220 complete datasets corresponding to each participant included in this study.

## Results

A description of the study cohort including the studied anthropometric parameters, height, age, BMR, average daily energy intake and relative daily energy intake variability, is presented in Table 1. The average values of the parameters listed in the table are presented with the corresponding standard deviation (x_s.d._, y_s.d._). For all the parameters, the range is also given, i.e., the minimum and maximum measured value. The statistical set presented in the table is divided into three groups. In the first part, all the participants are listed in summary. The second and third sections present statistics for men and women separately.

All the results of the multiple linear regression, i.e., regression coefficients, their standard deviations and statistical significance levels are presented in Table 2. Note that most of the regression coefficients listed in Table 2 are not dimensionless variables and have their units based on the units listed in Table 1. The results of the multiple linear regression presented in Table 2 show a statistically significant dependence of the percentage of body fat (p<0.01), BMI (p<0.01), and waist circumference (p<0.05) on the relative variability of the daily energy intake. The dependence of the waist-hip ratio on the relative variability of the energy intake was not statistically significant in our study (p>0.05). All the studied anthropometric parameters have statistically high significant dependence (p<0.001) on the independent parameters height, age and BMR. The anthropometric parameters BMI and percentage of body fat show similar dependence on the gender (p<0.001). The gender dependence of the anthropometric parameter waist circumference was also statistically significant (p<0.01).

The dependence of the waist-hip ratio on the gender was not statistically significant. The regression result also shows that the dependence of the anthropometric parameters on the average daily intake did not reach statistical significance (p>0.05) for any of the studied dependencies.

All the linear regression results are presented separately in Table 3 and Table 4 for men and women, respectively. In the case of the linear regression performed for women, a statistically significant relationship was found between the relative variability in the daily energy intake and BMI (p<0.05) and % body fat (p<0.05). The waist circumference and WHR were not statistically significant in the regression performed for women. In the case of the linear regression performed for men, no statistically significant dependence on the relative variability of the daily intake for any of the studied parameters (BMI, % BF, waist circumference and WHR) was found. Although the dependencies on the relative variability in the daily intake show larger values for the data obtained from men than from women, within standard deviations, these values are comparable, moreover, this dependence was not statistically significant for any of the anthropometric parameters for the men.

## Discussion

The aim of this study was to assess the effect of the variability in the daily energy intake observed over seven consecutive days on the total body fat and other anthropometric parameters in the study population. The study group consisted of approximately 70% women, although the recruitment of the participants was not limited by gender. This fact points to the trend of women being more concerned with their lifestyle and eating habits, which is not only related to the visual aspect, but also to health.

The study parameters, for which we assessed the dependence on the dietary variability, included the body fat percentage, BMI, waist circumference and WHR. They were chosen for their association with cardiometabolic disease risk.

The analysis of the results showed a statistically significant relationship between the body fat percentage (p<0.01), BMI (p<0.01) and waist circumference (p<0.05) and the variability in the energy intake. In contrast, the dependence of the waist and hip circumference ratio on the variability was found to be statistically insignificant (p=0.16).

Of all the anthropometric parameters studied, the % body fat was statistically found to be the most significantly dependent on the relative variability. According to the results of the regression coefficients, an increase in the variability by its standard deviation of 0.14 (corresponding to 14%) causes an average increase in body fat of 1.47%. The results of our study suggest that greater variability in the energy intake over seven consecutive days of the week was significantly related to a higher percentage of body fat. Comparable results were also reported by Tucker *et al.* (2000) 17, but they only observed this dependence in a group of middle-aged women with a BMI <30. However, in this study, a higher percentage of body fat was found to be associated with a higher overall energy intake. On the other hand, in our study, the average daily energy intake proved to be a statistically insignificant factor. These different conclusions could be due to the fact that the self-reporting may significantly underestimate the real energy intake, especially in obese women. In a 1992 study, the authors found that the selected study subjects underestimated the intake by an mean of up to 47±16 % 21.

In contrast, the results of another study 20 investigating the effect of the irregularity in the energy intake showed that despite relatively large fluctuations in the energy intake, there was a corrective response and the participants' weight remained stable despite these fluctuations. However, in this case, a small group of young women (n=15) with an average BMI of 22.2 showed greater variation in the energy expenditure than in the energy intake. The energy expenditure was not included in this study. However, according to a study by Bray *et al.* (2008), the biological mechanisms that regulate the energy and macronutrient intake are delayed by 3-4 days, so these biological signals may not be evident in short-term studies 19.

Fluctuations in the energy intake may occur most often on weekends 14,16,18. However, if a correction was made by reducing the energy intake on other days of the week, these fluctuations may not have had a significant effect on the sustained weight gain 16. The authors of the study explain this by saying that when people indulge in the foods they like at the weekend, it contributes to greater weight stability through a more sustainable lifestyle over the long term. In contrast, people who have a dichotomous view of eating, dividing foods into "do/don't" or "all or nothing" may not be as successful in losing or maintaining weight 16. However, in a study 18, the authors concluded that weekend lifestyle changes may instead be co-responsible for the gradual weight gain. However, it is not clear from this study whether these weight gains are due to an increase in the energy intake or due to the lower amount of physical activity.

The results of our study and other studies 17,18 suggest that the greater irregularity in the energy intake is likely to be associated with a higher percentage of body fat, provided there is inadequate compensation for the energy intake on the subsequent days. However, if this corrective response occurs and there is an adequate reduction in the energy intake after days of a higher energy intake, such fluctuations may not be associated with a higher body fat percentage, but instead may be considered a reasonable long-term sustainable dietary pattern 16. However, a longer-term study involving more related lifestyle factors would be needed to confirm these conclusions.

The second most statistically significant parameter of the studied anthropometric characteristics, which was dependent on the variations in the energy intake, appeared to be the body mass index (BMI). According to the results of the regression coefficients, an increase in the variability by its standard deviation of 0.14 causes an increase in the average BMI of 0.87. Although the use of BMI has its limitations, it is still the most used parameter to assess obesity. The fact that BMI together with % BF was found to be the most statistically significant factor due to the variability may indicate that the BMI generally correlates well with the % body fat 22. Nevertheless, for selected population groups, the use of BMI may not be appropriate because it does not differentiate between muscle and adipose tissue. Recognition accuracy in detecting an excess of fat mass is limited, particularly in men, where the BMI correlates better with the amount of fat-free mass. On the other hand, in women, the BMI appears to be a good diagnostic tool for detecting obesity defined by excessive body fat 22.

The last studied anthropometric parameter, the dependence of which proved to be statistically significant due to the relative variability in the energy intake, was the waist circumference. According to the results of the regression coefficients, an increase in the variability by its standard deviation of 0.14 causes an increase in the average waist circumference of 2.0 cm. The fact that the dependence on the variability was not as highly statistically significant as for the body fat percentage may be due to the fact that it is a parameter that tends to be treated differently in males and females. Nevertheless, the waist circumference can still be considered a very important parameter of anthropometric characteristics in humans, which is related to the amount of abdominal fat 23 and reflects well the lifestyle influence. A higher amount of visceral fat is associated with specific lifestyle factors 24, which may reflect dietary influences, including dietary regularity. Larger fluctuations in the food intake may be related, for example, to a lack of time and rest in order to prepare and eat a nutritionally rich and balanced diet.

The dependence of the WHR on the relative variability in the energy intake was not statistically significant. According to the results of the regression coefficients, when the variability increases by its standard deviation of 0.14, the average WHR increases by 0.0084. Considering that the dependence of the anthropometric parameters on the variability was demonstrated in all the other cases, we can conclude that the WHR does not exactly reflect a human's nutritional status. Besides genetic predisposition, the distribution of adipose tissue is also influenced by gender. However, the fact that WHR is not a reliable parameter indicating cardiometabolic risk is also demonstrated by the results of other studies 25,26. This is probably due to the fact that the WHR is not a reliable indicator of the amount of adipose tissue in the body, and therefore its use has recently been downgraded 27.

In the case of dividing the participants into two groups for men and women, a statistically significant dependence on the relative variability of the daily energy intake was found only in the group of women for the BMI and %BF values. In contrast to the whole study population, the values of the dependence of the waist circumference on the variability were not statistically significant for the female group. These different results could be explained by the higher prevalence of the gynoid body type of women. In the case of the data processed for men, none of these dependencies were statistically significant, despite the regression coefficients exhibiting higher values than the regression coefficients obtained from the group of women. This may be due to the smaller data set (69 men participated in the study compared to 151 women). The dependence of the WHR on the variability was not statistically significant for either separated group, which is consistent with the results of the analysis of the entire study population.

Although the variability in the food intake was found to be significantly associated with a higher body fat percentage, higher BMI and waist circumference in our study, we know, from many recent studies considering intermittent fasting, that, despite significant fluctuations in the energy intake, the excess body weight can be successfully reduced 28,29. This also goes against the general recommendation to reduce body mass through continuous energetic restriction.

These different results may be explained by the major difference in the attitude of individuals practicing a specific intermittent fasting protocol for weight reduction by targeted intervention. In contrast, people who have greater fluctuations in energy intake during a normal diet may have worse eating habits. These habits may include, for example, the less time spent preparing and eating food, emotional eating, skipping breakfast on weekdays, etc. Dietary habits may also reflect the quantity of high-quality protein, which has a higher thermic effect than other macronutrients 30 or the amount of dietary fiber consumed, which not only significantly influences fullness 31, but also regulates the amount of absorbed nutrients 32. Habitual short-term fasting or an inadequate energy intake can also result in increased energy use from the muscle mass. However, if fasting is consequently compensated, for example, at weekends, this may result in a gradual, slight increase in the amount of fat tissue at the expense of muscle mass. As the amount of muscle tissue decreases and the amount of adipose tissue increases, the basal metabolic rate may also decrease 33, which leads to the development of being overweight or obese over a longer period of time. However, still, the most important factor for higher amounts of adipose tissue is a long-term positive energy balance. Variability in energy intake may be only one of the many factors that affect energy balance. However, to confirm these findings, further longitudinal studies would be needed to analyze other dietary and lifestyle factors to bring them into context.

## Conclusion

Our research has demonstrated that people with a more regular energy intake have better values of their anthropometric characteristics related to cardiometabolic health, especially in the areas of body fat percentage, BMI and waist circumference. This may be related to the fact that people who have a more regular energy intake are also probably more likely to have better eating habits, including both better dietary composition and more time spent in preparing and consuming food. If people eat in a hurry, they do not invest enough time in preparing and eating their meals, which can result in changes in their eating habits. These facts can then lead to eating to catch up in the evening after work or at the weekend, when, in some cases, there is less physical activity. Long-term fluctuations in eating habits can result in gradual weight gain. Longer-term studies with larger numbers of participants are needed to confirm these findings.

## Figures and Tables

**Table 1:** Subject characteristics

	All (n=220)			Men (n=69)			Women (n=151)		
Variable	Mean	Standard Deviation	Range	Mean	Standard Deviation	Range	Mean	Standard Deviation	Range
**Body mass index (kg/m^2^)**	31.7	6.7	18.3-58	33.0	7.0	20.9-58	31.1	6.4	18.3-54.8
**Percentage of body fat (%)**	37.5	8.9	8.5-56	31.7	8.6	8.5-50.5	40.1	7.7	16.6-56
**Waist circumference (cm)**	104	18	61-161.1	111	17	80.1-159	101	18	61-161.1
**Waist-hip ratio**	0.94	0.10	0.66-1.22	0.978	0.071	0.82-1.15	0.92	0.11	0.66-1.22
**Height (cm)**	168.4	8.9	147.1-191.3	176.7	7.2	159.2-191.3	164.5	6.7	147.1-187.6
**Age (years)**	53	14	21.7-79.7	52	14	23.2-75.5	53	14	21.7-79.7
**Basal metabolic rate (kJ)**	6700	1200	4573-11712	7900	1200	5865-11712	6110	690	4573-8393
**Average energy intake (kJ/d)**	8800	2700	3560-25463	10400	2600	5611-19037	8100	2400	3560-25463
**Relative variability in daily energy intake**	0.23	0.14	0.045-1.546	0.216	0.087	0.088-0.566	0.23	0.16	0.045-1.546

**Table 2:** Regression coefficients b with standard deviations s.d. and statistical significance levels p (**all**)

	Body mass index			Percentage of body fat			Waist circumference			Waist-hip ratio		
	b	s.d.	p	b	s.d.	p	b	s.d.	p	b	s.d.	p
**Constant term**	57.7	9.0	<0.001	58	14	<0.001	92	26	<0.001	1.02	0.16	<0.001
**Height**	-0.486	0.056	<0.001	-0.392	0.087	<0.001	-0.75	0.16	<0.001	-38∙10^-4^	10 ∙10^-4^	<0.001
**Age**	0.144	0.024	<0.001	0.215	0.037	<0.001	0.561	0.070	<0.001	385∙10^-5^	44∙10^-5^	<0.001
**Basal metabolic rate**	655∙10^-5^	41∙10^-5^	<0.001	407 ∙10^-5^	64∙10^-5^	<0.001	148∙10^-4^	12∙10^-4^	<0.001	469∙10^-7^	75∙10^-7^	<0.001
**Average daily energy intake**	3∙10^-5^	14∙10^-5^	0.84	-22 ∙10^-5^	21∙10^-5^	0.30	19∙10^-5^	40∙10^-5^	0.62	35∙10^-7^	25∙10^-7^	0.16
**Relative variability in daily energy intake**	6.2	2.3	<0.01	10.5	3.6	<0.01	14.1	6.8	<0.05	0.060	0.042	0.16
**Gender**	3.96	0.95	<0.001	10.1	1.5	<0.001	7.9	2.8	<0.01	-0.020	0.017	0.25

**Table 3:** Regression coefficients b with standard deviations s.d. and statistical significance levels p (**men**)

	Body mass index			Percentage of body fat			Waist circumference			Waist-hip ratio		
	b	s.d.	p	b	s.d.	p	b	s.d.	p	b	s.d.	p
**Constant term**	86	19	<0.001	105	29	<0.001	143	45	<0.01	1.38	0.19	<0.001
**Height**	-0.62	0.11	<0.001	-0.67	0.17	<0.001	-1.05	0.26	<0.001	-56∙10^-4^	11∙10^-4^	<0.001
**Age**	0.147	0.052	<0.01	0.214	0.081	<0.05	0.60	0.13	<0.001	328∙10^-5^	55∙10^-5^	<0.001
**Basal metabolic rate**	594∙10^-5^	61∙10^-5^	<0.001	425∙10^-5^	94∙10^-5^	<0.001	140∙10^-4^	15∙10^-4^	<0.001	446∙10^-7^	63∙10^-7^	<0.001
**Average daily energy intake**	10∙10^-5^	24∙10^-5^	0.68	-14∙10^-5^	37∙10^-5^	0.70	46∙10^-5^	57∙10^-5^	0.43	38∙10^-7^	25∙10^-7^	0.13
**Relative variability in daily energy intake**	7.3	6.6	0.27	13	10	0.21	27	16	0.09	0.130	0.069	0.06

**Table 4:** Regression coefficients b with standard deviations s.d. and statistical significance levels p (**women**)

	Body mass index			Percentage of body fat			Waist circumference			Waist-hip ratio		
	b	s.d.	p	b	s.d.	p	b	s.d.	p	b	s.d.	p
**Constant term**	50.6	9.3	<0.001	50	15	<0.01	78	31	<0.05	0.87	0.21	<0.001
**Height**	-0.474	0.062	<0.001	-0.31	0.10	<0.01	-0.71	0.20	<0.001	-33∙10^-4^	14∙10^-4^	<0.05
**Age**	0.138	0.026	<0.001	0.209	0.042	<0.001	0.542	0.085	<0.001	402∙10^-5^	57∙10^-5^	<0.001
**Basal metabolic rate**	812∙10^-5^	59∙10^-5^	<0.001	487∙10^-5^	95∙10^-5^	<0.001	174∙10^-4^	19∙10^-4^	<0.001	55∙10^-6^	13∙10^-6^	<0.001
**Average daily energy intake**	5∙10^-5^	17∙10^-5^	0.78	-16∙10^-5^	28∙10^-5^	0.56	30∙10^-5^	56∙10^-5^	0.60	41∙10^-7^	38∙10^-7^	0.28
**Relative variability in daily energy intake**	5.3	2.6	<0.05	9.2	4.2	<0.05	10.4	8.4	0.22	0.042	0.056	0.46

## Abbreviations

BMI: body mass index
BMR: basal metabolic rate
WHR: waist-hip ratio
% BF: percentage of body fat
